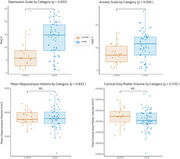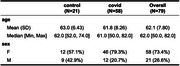# Mental Health and Brain Morphology: Insights from Long Covid cohort in underrepresented individuals

**DOI:** 10.1002/alz70857_106936

**Published:** 2025-12-26

**Authors:** João Pedro Uglione Da Ros, Lucas Uglione Da Ros, Andrei Bieger, Maiele Dornelles Silveira, Wyllians Vendramini Borelli, Joana Emilia Senger, Luiza Santos Machado, João Pedro Ferrari‐Souza, Marco Antônio De Bastiani, Guilherme Povala, Ana Paula Bornes da Silva, Guilherme Bastos de Mello, Arthur Viana Jotz, Matheus Fakhri Kadan, Graciane Radaelli, Daniele de Paula Faria, Artur Martins Coutinho, Mychael V. Lourenco, Tharick A Pascoal, Pedro Rosa‐Neto, Cristina Sebastião Matushita, Ricardo Benardi Soder, Artur Francisco Schumacher‐Schuh, Diogo O. Souza, Jaderson Costa da Costa, Débora Guerini de Souza, Eduardo R. Zimmer

**Affiliations:** ^1^ Universidade Luterana do Brazil, Canoas, RS, Brazil; ^2^ Universidade Federal Do Rio Grade Do Sul, Porto Alegre, Rio Grande do Sul, Brazil; ^3^ Masima: Macunaíma Soluções em Imagens Médicas, Porto Alegre, Rio Grande do Sul, Brazil; ^4^ Universidade Federal do Rio Grande do Sul, Porto Alegre, Rio Grande do Sul, Brazil; ^5^ Neurology Department, São Lucas Hospital of PUCRS, Porto Alegre, Rio Grande do Sul, Brazil; ^6^ Universidade Federal Rio Grande do Sul, Brazil, Porto Alegre, RS, Brazil; ^7^ Centro de Memória, Hospital Moinhos de Vento, Porto Alegre, RS, Brazil; ^8^ Brain Institute of Rio Grande do Sul (InsCer), PUCRS, Porto Alegre, Rio Grande do Sul, Brazil; ^9^ Universidade Federal do Rio Grande do Sul, Porto Alegre, RS, Brazil; ^10^ Department of Psychiatry and Neurochemistry, Institute of Neuroscience and Physiology, The Sahlgrenska Academy, University of Gothenburg, Gothenburg, VG, Sweden; ^11^ masima: Macunaíma Soluções em Imagens Médicas, Porto Alegre, Rio Grande do Sul, Brazil; ^12^ University of Pittsburgh, Pittsburgh, PA, USA; ^13^ Federal University of Rio Grande do Sul, Brazil, Porto Alegre, RS, Brazil; ^14^ Pontifícia Universidade Católica do Rio Grande do Sul, Porto Alegre, Rio Grande do Sul, Brazil; ^15^ Pontifícia Universidade Católica do Rio Grande do Sul, Porto Alegre, RS, Brazil; ^16^ Instituto do Cérebro do Rio Grande do Sul, Porto Alegre, RS, Brazil; ^17^ Universidade de São Paulo, São Paulo, SP, Brazil; ^18^ Universidade Federal do Rio de Janeiro, Rio de Janeiro, RJ, Brazil; ^19^ University of Pittsburgh School of Medicine, Pittsburgh, PA, USA; ^20^ McGill University Research Centre for Studies in Aging, Douglas Research Centre, Montreal, QC, Canada; ^21^ Hospital de Clínicas de Porto Alegre, Porto Alegre, RS, Brazil; ^22^ UFRGS, Porto Alegre, Brazil; ^23^ Brain Institute of Rio Grande Do Sul, PUCRS, Porto Alegre, RS, Brazil; ^24^ Federal University of Rio Grande do Sul (UFRGS), Porto Alegre, RS, Brazil

## Abstract

**Background:**

Neurological manifestations in individuals with Long COVID range from headaches to cognitive impairment and mental health issues. However, it remains unclear whether these individuals exhibit structural changes, functional changes, or both in the brain. In this study, we investigated the impact of Long COVID on mental health symptoms, cortical grey matter volume and thickness, and hippocampal volume in Brazilian individuals.

**Method:**

Individuals were divided into two groups based on Long COVID status: covid (symptoms of Long COVID) and control (No symptoms of Long COVID). Simultaneously, PHQ‐9 and GAD‐7 tests were applied on participants to evaluate severity of depression and generalized anxiety symptoms, respectively. Brain magnetic resonance imaging (MRI) of individuals presenting with Long COVID (*n* = 58) and of healthy control individuals (*n* = 21) were used for extracting volume and cortical thickness (CT) of regions of interest using FreeSurfer (v7.4.1). We performed an ANCOVA analysis and a linear regression to assess the difference between groups in PHQ‐9, GAD‐7, mean cortical thickness (CT), mean hippocampal volume, and total cortical grey matter volume. The data were corrected for age, sex, and years of formal education.

**Result:**

The Long Covid group presented significantly lower scores on PHQ‐9 and GAD‐7 than the control group (Beta = 6.20017 and 3.3105; *p* <0,001 and *p* <0,006, respectively). However, when comparing Long covid and control groups, we found no significant differences in the mean hippocampal volume (*p* = 0,831) and in the mean cortical grey matter volume (*p* = 0.193).

**Conclusion:**

These preliminary data indicate significant changes in mental health among individuals with Long Covid; however, these changes do not correspond to observable alterations in brain volume as seen in MRI scans. This suggests that the pathophysiological changes associated with these symptoms are likely functional and metabolic in nature rather than structural and may not be detectable through imaging studies that primarily focus on brain anatomy, such as MRI.